# Changes in thyroid hormones, leptin, ghrelin and, galanin following oral melatonin administration in intact and castrated dogs: a preliminary study

**DOI:** 10.1186/s12917-019-1894-9

**Published:** 2019-05-14

**Authors:** Pegah Taheri, Asghar Mogheiseh, Aidin Shojaee Tabrizi, Saeed Nazifi, Sina Salavati, Farzaneh Koohi

**Affiliations:** Department of Clinical Sciences, School of Veterinary Medicine, Shiraz University, P.O.Box 71441-69155, Shiraz, Fars Iran

**Keywords:** Castration, Dog, Galanin, Ghrelin, Leptin, Melatonin, Thyroid hormones

## Abstract

**Background:**

Melatonin regulates metabolism and metabolism related hormones in mammalians. Castration has some adverse effects on the metabolic hormones of dog. This study was conducted to determine the effects of oral melatonin administration on metabolic hormones, as well as to compare changes of these hormones after administration of melatonin in castrated and intact dogs. Twenty healthy mixed breed mature male dogs were divided randomly into four groups (*n* = 5): melatonin (3 mg/10 kg(, castrated, castrated and melatonin treated, and negative control. Blood sample was collected from jugular vein weekly for 1 month.

**Results:**

T3 and T4 hormones had a significant decrease within 1 month following administration of melatonin. No significant change was observed in concentration of FT3 and FT4 hormones. Leptin and ghrelin hormones also had a significant decrease in this period. Leptin and ghrelin had a more significant decrease in “non-castrated and melatonin treated” group compared to “castrated and melatonin treated” group. Galanin had a significant decrease but this neurotransmitter had no significant change in “non-castrated and melatonin treated” group in comparison to “castrated and melatonin treated” group.

**Conclusions:**

It seems that daily administration of melatonin capsule in all dogs can probably decrease concentration of T3 and T4 hormones and balance other metabolic hormones following castration.

**Methods:**

The dogs underwent castration, melatonin treatment and blood sampling.

## Background

Castration in dogs is commonly performed due to its benefits including contraception, population control, prevention of reproductive disorders and undesired sexual behavior. However, anesthesia and surgical complications, increased risk of prostate cancer, increased musculoskeletal and endocrine disorders and obesity have been considered as castration disadvantages [1]. Obesity as the most common nutritional disorder has become a growing concern in neutered dogs. Decreased metabolic rate, increased food intake and decreased activity are recognized as reasons of obesity following castration [2]. There is no pharmaceutical drug used in dogs to treat or prevent obesity. Nevertheless, melatonin hormone is known as a key mediator that can regulate energy balance and body weight [3].

Melatonin (N-acetyl-5-methoxy tryptamine) is secreted from pineal gland in darkness and regulates mammalian reproduction and secretion of endocrine glands [4]. Other sources of melatonin are neuroendocrine cells in retina and thyroid gland and peripheral tissues such as pancreas, gastrointestinal tract and immune cells [5]. Pineal gland is recognized as a neuroendocrine transducer, it means that this gland can transform seasonal changes to nervous impulses; these impulses regulate endocrinal responses in animals with seasonal reproduction [6]. Melatonin is made of tryptophan amino acid after undergoing several enzyme reactions. Melatonin has antioxidant, anti-aging and anti-stress effects. It also can regulate immune system, physiologic rhythms, sleeping and gonadotrophic function especially in seasonal breeding animals. However, there are few documents on melatonin’s role in non-seasonal breeder animals like dogs. Thyroid hormones (T3 and T4), leptin, ghrelin and galanin have an important role in body metabolism.

Thyroxin (T4) and triiodothyronine (T3) are secreted from thyroid gland, both of them increase metabolic rate in body. Lack of thyroid hormones secretion in body can decrease body metabolism to 40–50% less than normal [7]. A more than usual decrease in thyroid hormones almost always leads to an increase in body weight [6]. Melatonin is secreted from pineal gland and thyroid c-cells under thyroid stimulating hormone, so melatonin can affect thyroid growth and/or function as an endocrine and paracrine hormone [8]. The decrease of thyroid hormone circulating levels is described after melatonin administration [9]. Melatonin influenced thyroid hormones levels by controlling iodothyronine-deiodinases. Melatonin has an inhibitory effect on cell proliferation and thyroid hormone synthesis and a protective effect against oxidative damage in the thyroid gland [10–12]. The inhibition of thyroid growth and/or thyroid function by the pineal gland, as well as the stimulation of the pineal gland activity and growth processes by the thyroid hormones, have prompted a hypothesis on the existence of a reciprocal relationship between the thyroid and the pineal [13].

Leptin is a hormone secreted from adipose tissue [14]. Leptin is ghrelin’s antagonist and reduces food intake [6] and body weight [15]. We should consider that melatonin receptors have been discovered in adipose tissue where leptin is produced [16]. Ghrelin is a signal peptide secreted when an animal is hungry. Feeding reduces ghrelin levels in plasma [17]. This hormone is secreted from gastrointestinal tract and hypothalamus. On the other hand, we should consider that stomach and hypothalamus are two of the places containing melatonin receptors [18].

Leptin can directly regulate melatonin secretion by its effect on pineal gland [19]. Fasting increases plasma ghrelin and decreases plasma leptin, after feeding, ghrelin levels start to decrease and leptin levels increase. Leptin is actually considered an anorexigenic hormone [20]. In a study performed on Siberian hamster, exogenous melatonin reduced plasma leptin [20]. It is important to consider that leptin changes are long-term but ghrelin is a fast acting hormone [14]. Leptin is known to have a stimulatory effect on T3 and T4 secretion in sheep; however the effect of melatonin is dependent on the season. Melatonin decreased T4 levels in long and short days but, in long days’ photoperiod, melatonin increased T3 and in short photoperiods decreased T3 concentrations [19].

Galanin is a neurotransmitter that regulates different physiologic functions including gastrointestinal muscle contractions, insulin inhibition, comprehension, sleeping, eating, behavior and blood pressure [21]. Galanin displays inhibitory effects in the endocrine pancreas and, like melatonin, reduces insulin secretion in β-cells by binding to Gαi-coupled receptors (GALR1–3) [22]. Galanin is associated with many diseases like Alzheimer, leprosy, depression, gastrointestinal disorders and cancer [23, 24].

In this study we want to realize the effects of oral melatonin on levels of thyroid hormones, leptin, ghrelin and galanin in castrated and non-castrated dogs and determine whether oral melatonin administration can be effective in balancing the most common castration induced metabolic hormone disturbances.

## Methods

### Animal ethics

All animal experiments were approved by the State Committee on Animal Ethics, Shiraz University, Shiraz, Iran (IACUC no: 4687/63). The recommendations of European Council Directive (2010/63/EU) of September 22, 2010 regarding the standards in the protection of animals used for experimental purposes were also followed.

### Animals and experimental design

**Fig. 1:**
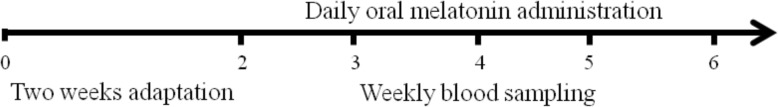
Schema of study design, dogs were adapted with new condition and dewormed during first 2 weeks. Oral melatonin was administrated immediately after castration time and blood sample was collected from jugular vein weekly and from 2 days after day of castration at 10 a.m

All dogs were stray or free-roaming. Under a population control program with cooperation of nongovernmental organizations (NGOs) shelters, they were captured and spayed at the end of study. Twenty intact mixed breed male dogs with age of 1–3 years old and mean body weight of 20 kg were selected for this study. During 2 weeks of preparation, the dogs were dewormed (Praziquantel; one tablet for 10 kg/BW; Mebendazole; one tablet for 5 kg/BW). They received 12-h light, 12-h dark lighting schedule that supplemented the natural daylight and 300 g of commercial dog food daily (NUTRI Dry Dog Food; Behintash Co. Iran). Then, the dogs were randomly divided into four groups in a case-control pilot study. The first group, intact dogs, received 3 mg/10 kg BW of melatonin (L’ORGANIQUE, Canada) orally daily for 1 month, the second group, castrated dogs, did not receive melatonin, the third group, castrated dogs, received 3 mg/10 kg BW of melatonin orally daily for 1 month and the fourth group, false castrated dogs, used as negative control, received melatonin vehicle. Dogs in negative control were manipulated as castrated dogs but were not castrated. Melatonin vehicle was just empty gelatinous capsule. The dose for oral melatonin administration was 3 mg/10 kg BW and administrated immediately after castration time in melatonin and castrated melatonin treatment groups. Blood sample was collected from jugular vein weekly at 10 a.m. (first sample from all dogs: Two days after day of castration = day 0 of sampling) for 1 month for determination of serum concentration of thyroid hormones, leptin, ghrelin and galanin (Fig. 1). Blood samples were sent to laboratory in glass tubes. Sera were extracted using 750 *g* centrifugation for 10 min within 2 h following sampling and were kept in a − 20 °C refrigerator.

### Castration method

Ten dogs were selected randomly for castration. Anesthesia premedication was done using Acepromazine (0.05 mg/kg, IM, Alfasan, Woerden, Holland) and Morphine (0.1 mg/kg, IM, Darou Pakhsh, Iran). Induction was performed using Ketamine (5 mg/kg, IV, Alfasan, Woerden, Holland) and Midazolam (0.2 mg/kg, IV, Darou Pakhsh, Iran). Isoflurane (1.2%, Nicholas Piramal Limited, London. UK) was used as maintenance inhalation drug. After induction of anesthesia, one of the testes was presented to pre-scrotal area by pressing through the scrotal region, an incision was made over the testis. Then spermatic fascia and tunica vaginalis were incised. A hemostat was placed in the spot where tunic attaches to epididymis and ligament of tail of epididymis was separated using fingers. Both ductus deferens and vascular cord were ligated individually, and then both were ligated together by circumferential ligature. A hemostat was placed below the ligatures and the space between hemostat and ligatures was transected [25].

### Measurement of the parameters

Serum T3 and T4 were measured using Iran Padtan-Elm competitive ELISA kit with sensitivity of 0.5 ng/ml and 0.4μg/dl respectively. FT3 and FT4 were measured using USA Monobind Competitive ELISA kit with sensitivity of 0.835 pg/ml and 0.314 ng/dl respectively. Serum leptin, ghrelin and galanin levels were measured using a solid phase sandwich ELISA method (dog ELISA Kits; Bioassay Technology Laboratory, Shanghai Crystal Day Biotech Company, China).

### Statistical analysis

Results were analyzed by One-way repeated measures ANOVA and Tukey’s multiple comparisons test to compare overall mean concentration of each hormone between groups and Two-way repeated measures ANOVA method with matching both factors and their interactions (Time: 0, 7, 14, 21 and 28 and Group: Melatonin, Castrated, Mel+Cast and Control) using Tukey’s multiple comparisons test by Graph Pad Prism7 software. The differences were considered significant when *P* value was less than 0.05.

## Results

### T3

**Fig. 2:**
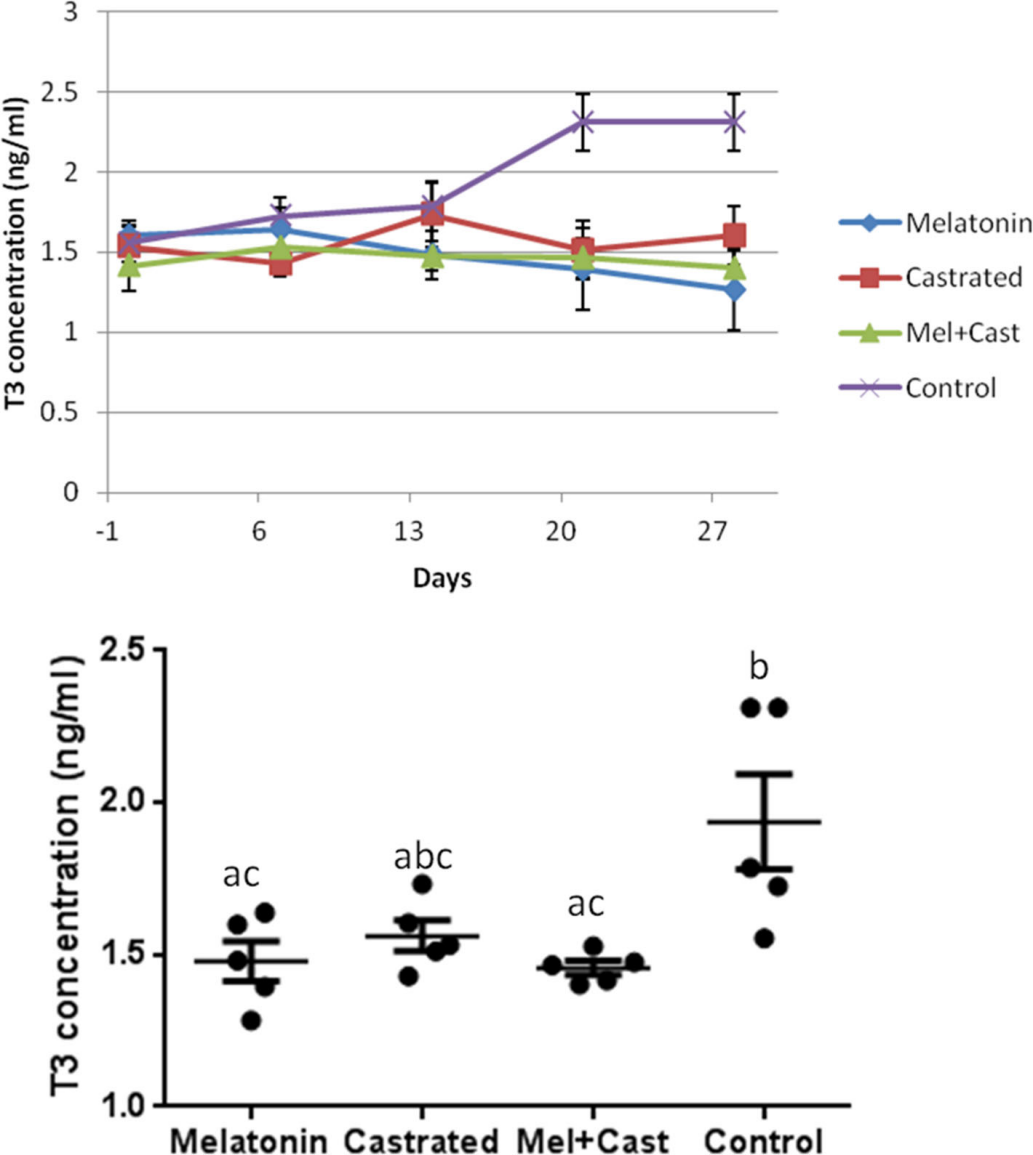
Changes and comparison of overall means±SEM concentration of T3 hormone between control, melatonin, castrated and melatonin+castrated groups. Each black round point indicates mean concentration of T3 hormone of each dog during study. Different letters indicate significant differences between groups (*P* < 0.05)

The mean concentration of T3 hormone in control group was 1.93 ± 0.15 ng/ml, in castrated dogs 1.56 ± 0.05, in melatonin treated dogs 1.47 ± 0.06 and in castrated and melatonin treated dogs 1.45 ± 0.02. Significant difference was observed in mean concentration of T3 among all groups (*P* = 0.008) and between control vs. melatonin (*P* = 0.01) and control vs. melatonin+castrated (P = 0.01) groups, but time factor did not affect T3 concentration. At day 21 and 28, differences were significant between control vs. melatonin (*P* < 0.0001), control vs. castrated (*P* < 0.004) and control vs. melatonin+castrated (*P* < 0.0002). When T3 levels were analyzed in each group among different sampling times, there were significant differences between time 0 vs time 21 and time 0 vs. time 28 (*P* = 0.001), time 7 vs. time 21 and time 7 vs. time 28 (*P* = 0.01) and time 14 vs time 21 and time 14 vs. time 28 (*P* = 0.04) in control group (Fig. 2).

### T4

**Fig. 3:**
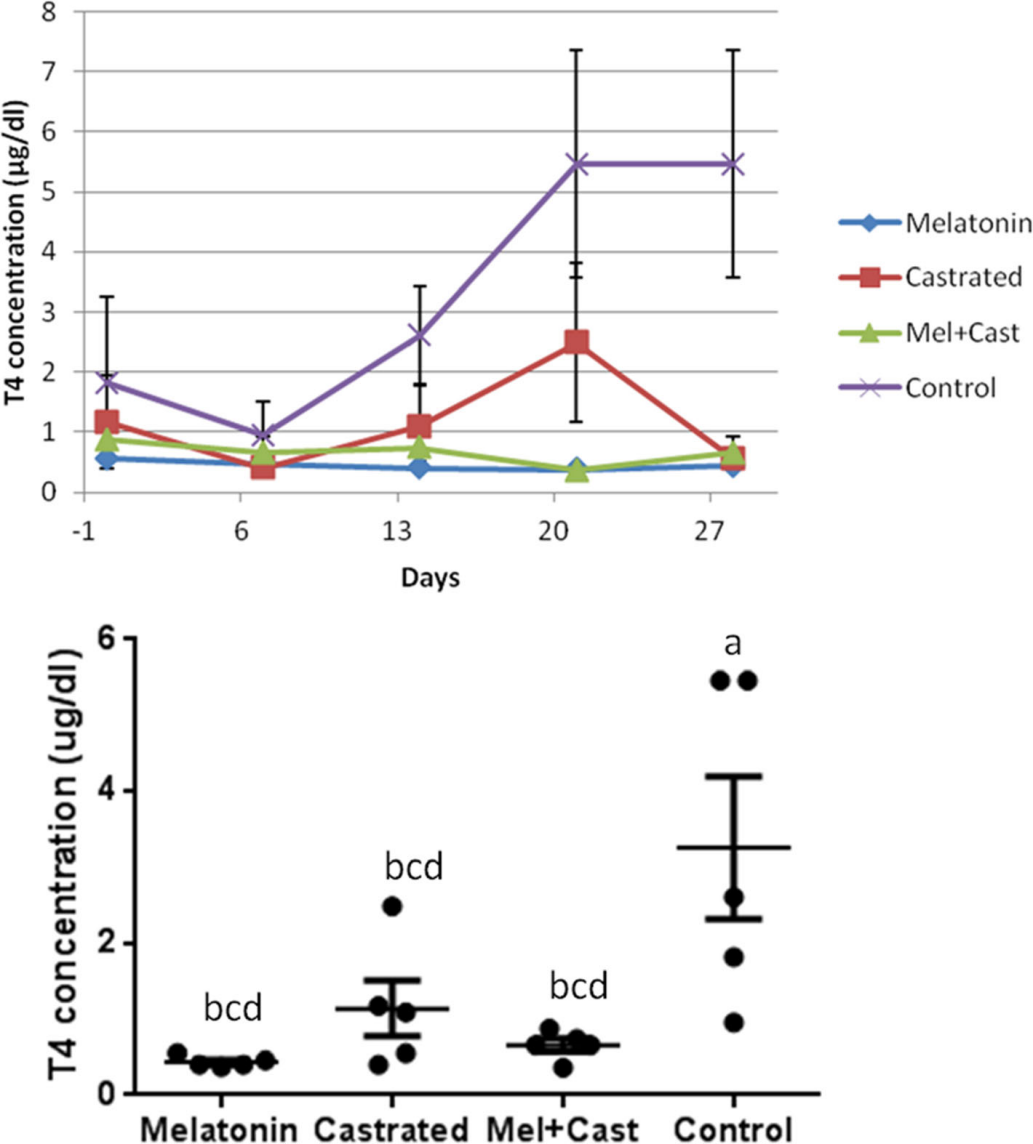
Changes and comparison of overall means±SEM concentration of T4 hormone between control, melatonin, castrated and melatonin+castrated groups. Each black round point indicates mean concentration of T4 hormone of each dog during study. Different letters indicate significant differences between groups (*P* < 0.05)

The mean concentration of T4 hormone in control group was 3.26 ± 0.70 mg/dl, in castrated dogs 1.14 ± 0.36, in Melatonin treated dogs 0.44 ± 0.03 and in castrated and melatonin treated dogs 0.65 ± 0.03 mg/dl. Overall concentration of T4 was significantly different among groups (*P* = 0.0004), between control vs. melatonin (*P* = 0.0005), control vs. castrated (*P* = 0.005) and control vs. melatonin+castrated (*P* = 0.001). There were significant differences in mean levels of T4 between control vs. melatonin and control vs. melatonin+castrated groups at day 21 (*P* = 0.0002) and at day 28 between control vs. melatonin (*P* = 0.0003), control vs. melatonin+castrated (*P* = 0.0005) and control vs. castrated (*P* = 0.0004) groups. When changes of T4 levels during 4 weeks were analyzed in each group, there were significant changes between day 0 vs day 21 and day 0 vs 28 (*P* = 0.01) and day 7 vs. day 21 and day 7 vs. 28 (*P* = 0.001) in control group (Fig. 3).

### FT3

**Fig. 4:**
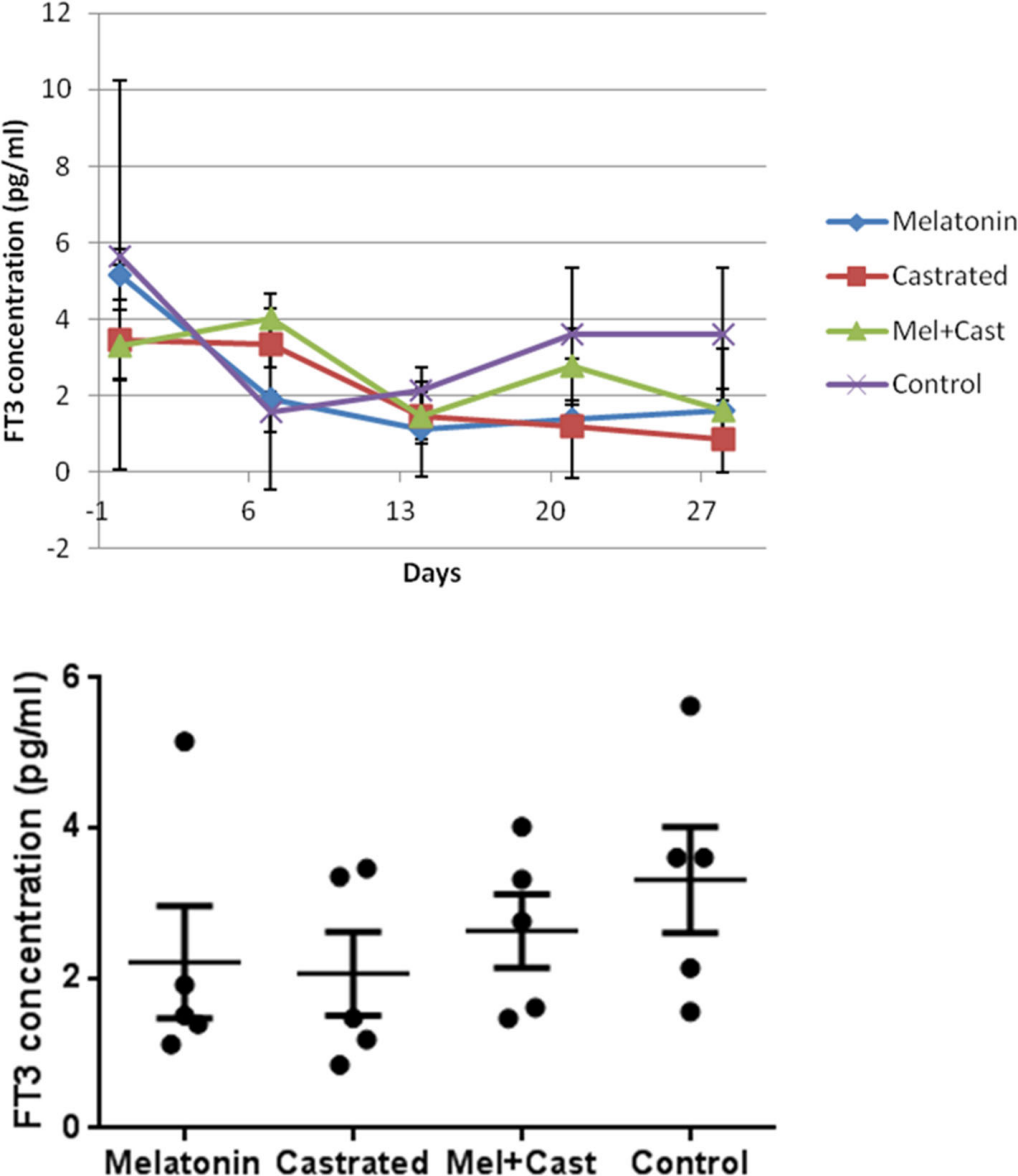
Changes and comparison of overall means±SEM concentration of FT3 hormone between control, melatonin, castrated and melatonin+castrated groups. Each black round point indicates mean concentration of FT3 hormone of each dog during study

The mean concentration of FT3 hormone in control group was 3.30 ± 0.81 pg/ml, in castrated dogs 2.06 ± 0.55, in melatonin treated dogs 2.23 ± 0.74 and in castrated and melatonin treated dogs 2.63 ± 0.49 pg/ml. In statistical analysis, there were not any significant differences in FT3 concentration among all groups and between them (*P* = 0.38). But, time factor (*P* = 0.001) affected FT3 levels and there were significant differences between control vs. melatonin+castrated groups at day 0 (*P* = 0.04), control vs. melatonin+castrated groups at day 7 (*P* = 0.02), control vs. castrated groups at day 21 (*P* = 0.03) and control vs. castrated groups at day 28 (*P* = 0.01). Moreover, analysis of changes of FT3 levels during 4 weeks in each group revealed that there were significant differences in melatonin group between day 0 vs. day 7 (*P* = 0.003), day 0 vs. day 14 (*P* = 0.0002), day 0 vs. day 21 (*P* = 0.0005) and day 0 vs. day 28 (*P* = 0.001), in castrated group between day 0 vs. 28 (*P* = 0.02) and day 7 vs. 28 (*P* = 0.03), in melatonin+castrated group between day 7 vs. day 14 (*P* = 0.03) and in control group between day 0 vs. day 7 (*P* = 0.0002) and day 0 vs. day 14 (*P* = 0.001; Fig. 4).

### FT4

**Fig. 5:**
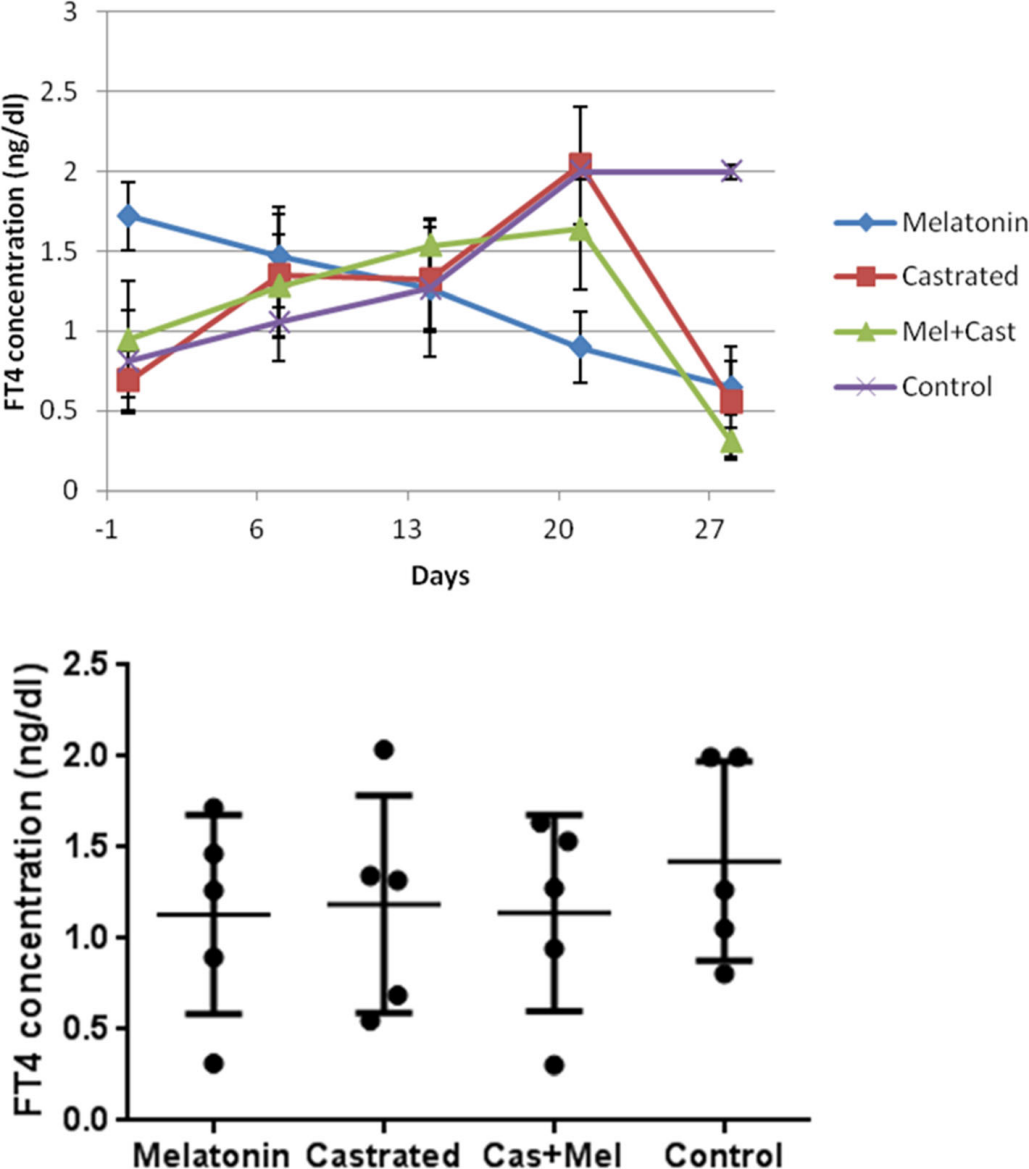
Changes and comparison of overall means±SEM concentration of FT4 hormone between control, melatonin, castrated and melatonin+castrated groups. Each black round point indicates mean concentration of FT4 hormone of each dog during study

The mean concentration of FT4 hormone in control group was 1.42 ± 0.22 ng/dl, in castrated dogs 1.18 ± 0.26, in melatonin treated dogs 1.19 ± 0.24 and in castrated and melatonin treated dogs 1.14 ± 0.24 ng/dl. There was no significant difference in concentrations of FT4 between groups (*P* = 0.55). But, time factor (*P* = 0.01) affected FT4 levels and there were significant differences between castrated vs. melatonin group at day 0 (*P* = 0.03), castrated vs. melatonin and control vs. melatonin groups at day 21 (*P* = 0.01) and control vs. melatonin (*P* = 0.02), control vs. castrated (*P* = 0.001) and control vs. melatonin+castrated (*P* = 0.0001) groups at day 28. Moreover, analysis of changes of FT4 levels during 4 weeks in each group revealed that there were significant differences in melatonin group between day 0 vs. 28 (*P* = 0.03), castrated group between day 0 vs. day 21 (*P* = 0.004) and day 21 vs. day 28 (*P* = 0.001), melatonin+castrated group between day 14 vs. day 28 (*P* = 0.01) and day 21 vs. day 28 (*P* = 0.005) and control group between day 0 vs. day 21 and day 0 vs. day 28 (*P* = 0.01; Fig. 5).

### FT3/FT4 ratio

**Fig. 6:**
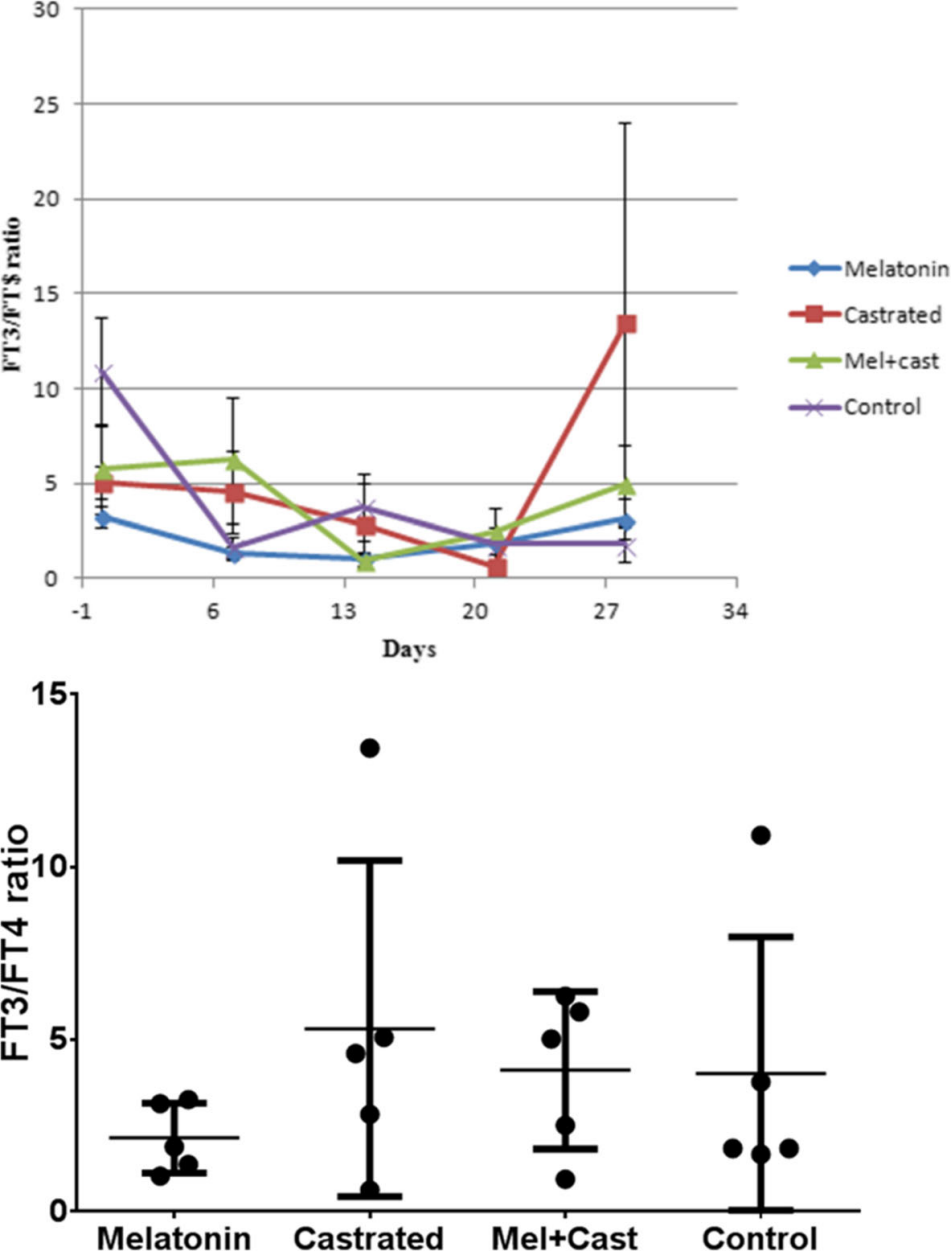
Changes and comparison of overall means±SEM ratio of FT3/FT4 between control, melatonin, castrated and melatonin+castrated groups. Each black round point indicates mean concentration of FT3/FT4 ratio of each dog during study

In general, there was no significant difference between groups. At day 28 of sampling, significant differences were observed between castrated vs. melatonin groups (*p* = 0.03), castrated vs. control groups (*p* = 0.01) and control vs. melatonin and castrated groups (*p* = 0.009). In castrated group, significant differences were revealed between day 28 of sampling and day 14 (*p* = 0.03) and 21 (*p* = 0.007; Fig. 6).

### Leptin

**Fig. 7:**
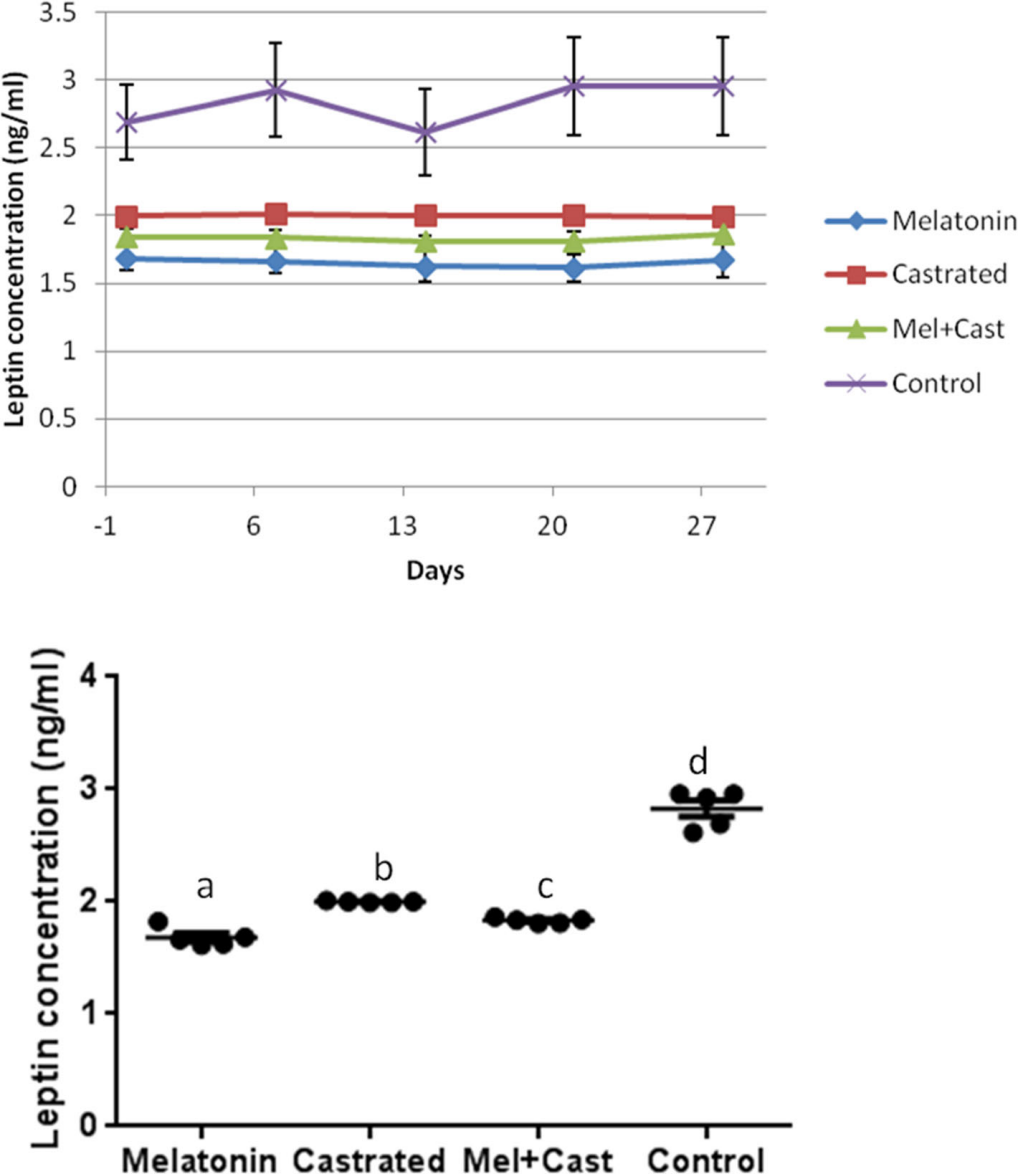
Changes and comparison of overall means±SEM concentration of Leptin hormone between control, melatonin, castrated and melatonin+castrated groups. Each black round point indicates mean concentration of Leptin hormone of each dog during study. Different letters indicate significant differences between groups (*P* < 0.05)

The mean concentration of leptin hormone in control group was 2.83 ± 1.01 ng/ml, in castrated dogs 2 ± 0.003, in melatonin treated dogs 1.65 ± 0.03 and in castrated and melatonin treated dogs’ 1.83 ± 0.01 ng/ml. Overall concentrations of leptin were significantly different among groups (*P* < 0.0001) and between castrated vs. melatonin (*P* = 0.02), control vs. melatonin, control vs. castrated and control vs. melatonin+castrated groups (*P* < 0.0001). Moreover, multiple comparisons of leptin levels between groups in each sampling time revealed significant differences between control vs. melatonin (*P* = 0.0009), control vs. castrated (*P* = 0.03) and control vs. melatonin+castrated (*P* = 0.006) groups at day 0, control vs. melatonin (*P* < 0.0001), control vs. castrated (*P* = 0.002) and control vs. melatonin+castrated (*P* = 0.0003) groups at day 7, control vs. melatonin (*P* = 0.001) and control vs. melatonin+castrated (*P* = 0.01) groups at day 14, control vs. melatonin (*P* < 0.0001), control vs. castrated (*P* = 0.001) and control vs. melatonin+castrated (*P* = 0.0001) groups at day 21 and control vs. melatonin (*P* < 0.0001), control vs. castrated (*P* = 0.001) and control vs. melatonin+castrated (*P* = 0.0003) groups at day 28. There were not any significant differences between sampling times in each group (Fig. 7).

### Ghrelin

**Fig. 8:**
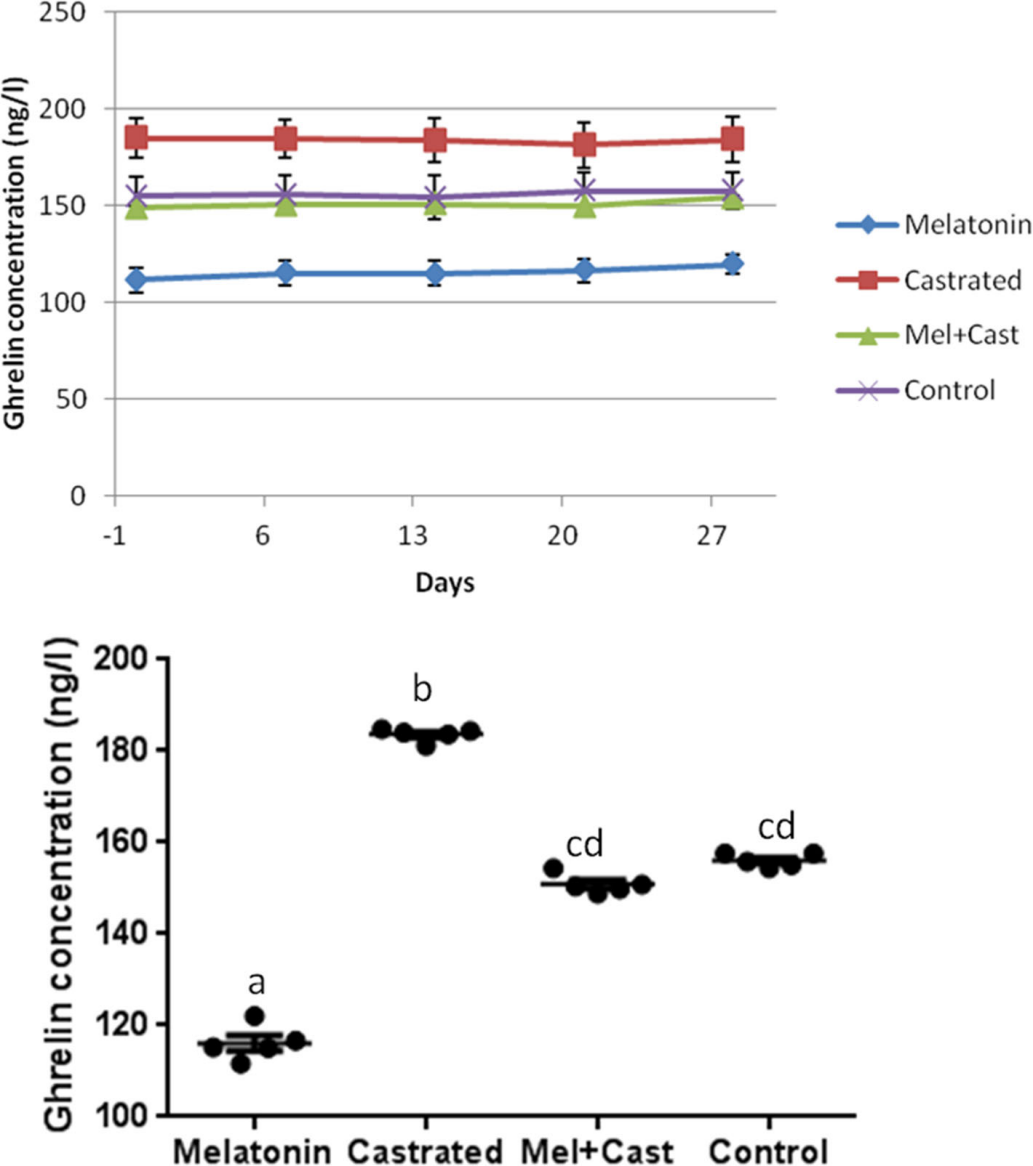
Changes and comparison of overall means±SEM concentration of Ghrelin hormone between control, melatonin, castrated and melatonin+castrated groups. Each black round point indicates mean concentration of Ghrelin hormone of each dog during study. Different letters indicate significant differences between groups (*P* < 0.05)

The mean concentration of ghrelin hormone in control group was 156.1 ± 0.78 ng/l, in castrated dogs 183.6 ± 0.63, in melatonin treated dogs 115.6 ± 1.69 and in castrated and melatonin treated dogs 150.8 ± 0.95 ng/l. Overall comparison of mean concentration of ghrelin revealed significant differences among groups (*P* < 0.0001) and between melatonin vs. castrated (*P* < 0.0001), melatonin vs. melatonin+castrated (*P* = 0.005), melatonin vs. control (*P* = 0.001), castrated vs. melatonin+castrated (*P* = 0.008) and control vs. castrated (*P* = 0.02) groups. Analysis of mean concentration of ghrelin between groups in each time of sampling revealed significant differences between all groups (*P* < 0.03) except control vs. melatonin+castrated group. But, there were not any significant differences in ghrelin levels between days of sampling in each group (Fig. 8).

### Galanin

**Fig. 9:**
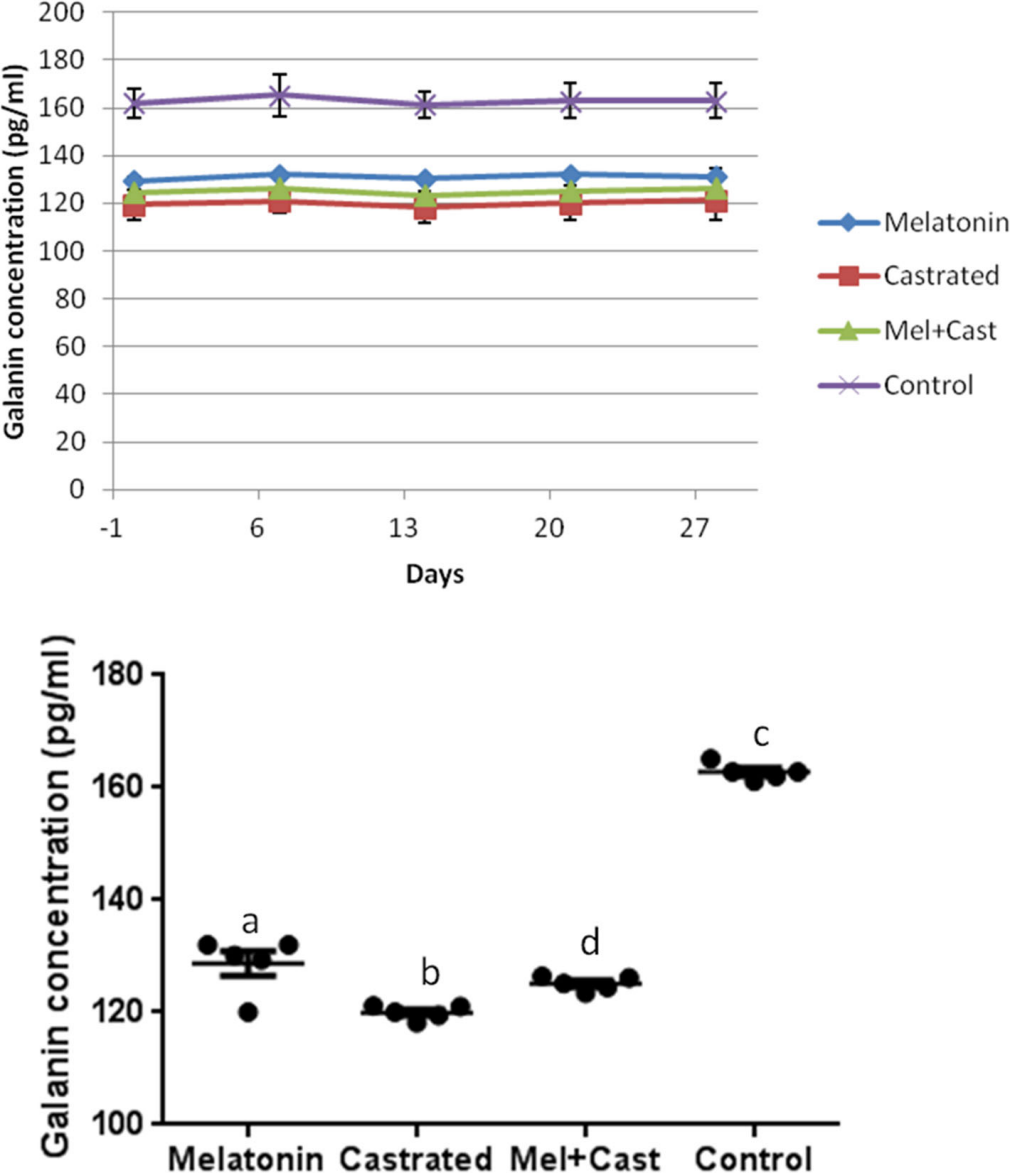
Changes and comparison of overall means±SEM concentration of Galanin hormone between control, melatonin, castrated and melatonin+castrated groups. Each black round point indicates mean concentration of Galanin hormone of each dog during study. Different letters indicate significant differences between groups (*P* < 0.05)

The mean concentration of galanin hormone in control group was 162.8 ± 0.39 pg/ml, in castrated dogs 120 ± 0.54, in melatonin treated dogs 131 ± 2.23 and in castrated and melatonin treated dogs 125.1 ± 0.56 pg/ml. There was significant effect of group factor among all groups (*P* < 0.0001) and between control vs. melatonin (*P* = 0.0005), control vs. castrated (*P* < 0.0001) and control vs. melatonin+castrated (*P* = 0.0001) groups. Also, statistical analyses of galanin levels between groups in each sampling day revealed significant differences between control vs. melatonin, control vs. castrated and control vs. melatonin+castrated groups in each day of sampling (*P* < 0.0004). But, there were not any significant differences in comparison of mean concentration galanin between different days of sampling in each group (Fig. 9).

## Discussion

### T3, T4, FT3, FT4 and FT3/FT4

Results of the present study revealed that T3 and T4 decreased following castration. Oral administration of melatonin decreased T3 and T4 levels in intact dogs. So, in castrated dogs that were treated with melatonin, concentration of T3 level was lower than control and similar to melatonin group, T4 level was lower than intact dogs and higher than melatonin and non-castrated dogs.

In this study castration in male dogs decreased serum T4 concentration. This is probably the reason why diminished metabolism is observed in castrated dogs. A study revealed epidemiologic feature of canine hypothyroidism such that risk of hypothyroidism increased in both gonadectomized male and female dogs [26]. Decreased relative volume densities of the follicles, colloid and epithelium and increased interstitium of thyroid gland were observed in castrated dogs compared to intact dogs. This effect was reversible with chronic treatment with testosterone propionate [27]. Also, oral melatonin administration in 4 weeks decreased T3 and T4 concentration in intact dogs; this may be due to melatonin’s role in metabolism reduction. Oral melatonin administration in castrated dogs for 4 weeks had a more intense effect on T3 and T4 decrement in comparison to the control group; the probable reason for this finding is that castration and melatonin administration had synergistic effect on reducing T3 and T4 levels. Previous studies about rodents [28] and foxes [29] had the same results and oral melatonin administration in these studies also caused a decrement in thyroid hormones. Other researchers did not show any changes in thyroxin concentrations following oral administration of 1–1.3 mg/kg melatonin in female and male intact dogs [30]. In present study, FT3 and FT4 hormones had no significant change; possibly due to the fact that we did not use equilibrium dialysis as the method of choice for detection of these parameters [31] and instead, they were measured by ELISA as the only available method. The other reason why no significant change was observed in FT3 and FT4 levels may be because the test measures free hormone levels which are very small in amount and more than 99.5% of thyroid hormones are bound to protein so this test is technically more difficult than measuring T4 [6]. Considering the fact that oral melatonin administration reduced T3 and T4 levels, melatonin may have potential to be used for treatment of hyperthyroidism along with other drugs such as methimazole; however, this hypothesis needs further investigations.

### Leptin and ghrelin

This study’s results revealed that oral melatonin administration in castrated dogs causes a significant decrease in serum leptin levels. Also, castration reduced leptin levels in comparison to intact dogs, this may be because in castration both testes which contain adipose tissue are removed and leptin is secreted from adipose tissue [14]. Oral melatonin administration in castrated dogs made serum leptin decrease significantly in comparison to the castrated group. In previous studies, after long term oral administration of melatonin in rats, bodyweight and plasma leptin concentration were reduced [32]. Also, in another study melatonin administration (1 g for each rat) reduced leptin levels (at 23p.m) [33]. In Rasmussen et al.’s study in 1999 exogenous melatonin administration caused a reduction in leptin levels of rat and weasel blood. The results were similar in all previously mentioned studies [34].

Results also showed that in oral melatonin administration group serum ghrelin level was significantly lower than castrated group (*P* < 0.0001). This may be because Melatonin administration decreases appetite and ghrelin level subsequently, but castration causes an increase in appetite and ghrelin. Also in melatonin administration group serum ghrelin level was significantly lower than the castrated and melatonin group (*p* < 0.0001), this may be because in the second group, castration increases serum ghrelin level and covers melatonin effect. The melatonin group had a significant decrease in ghrelin level in comparison to control group (*P* < 0.0001). In castration and melatonin group serum ghrelin level was significantly lower than the castrated group (*P* < 0.0001), this result is probably because melatonin’s effect on ghrelin decrement in the first group covers castration effect on ghrelin increase. In the castrated group serum ghrelin level was significantly higher than the control group (*P* < 0.0001). Serum ghrelin level comparison between the control group and the castrated and melatonin group was insignificant. After castration male dog’s ghrelin level increased, however, in intact dogs after melatonin administration ghrelin level decreased dramatically. One of the most important findings of this study is that increased level of ghrelin following castration can be reduced and similar the intact dog’s ghrelin level by oral melatonin utilization thus oral melatonin administration (3 mg/10kgBW) is likely useful in preventing and controlling obesity after castration. In another study in rat, exogenous melatonin administration destroyed the negative correlation between plasma ghrelin and leptin levels [32], this may be due to ghrelin suppression by exogenous melatonin. Also, early study revealed that plasma ghrelin concentration in rats will decrease significantly after exogenous melatonin administration; all these facts match our study results.

### Galanin

In this study, serum galanin level in the castrated group was significantly lower than the melatonin group (*p* = 0.0008) and the highest decrease in galanin level was in the castrated group. Melatonin administration in intact dogs caused a significant decrease in galanin concentration. Melatonin administration in castrated dogs produced a significant decrease in galanin; however, this decrement was not as intense as melatonin administration in intact dogs. In a study that was done by Naftalin et al. in 2004, it was shown that both melatonin and galanin can have an inhibitory effect on glucose transmission to brain [35]. Also, higher than normal levels of melatonin in conditions such as blindness or exogenous melatonin consumption cause an increase in blood glucose level [36]. Blood glucose increase after galanin infusion in dogs is also reported but such an effect has not been significant in humans [37]. Nevertheless, there is no literature conducted on the direct effect of melatonin on galanin so far and these results are reported for the first time. Considering the fact that melatonin administration leads to drop in galanin, we can consider the probable effect of melatonin in controlling neurologic diseases such as leprosy and canine cognitive dysfunction. In these diseases galanin levels increase because of neuronal damage. However, this is a hypothesis and should be proved in further studies.

**Fig. 10:**
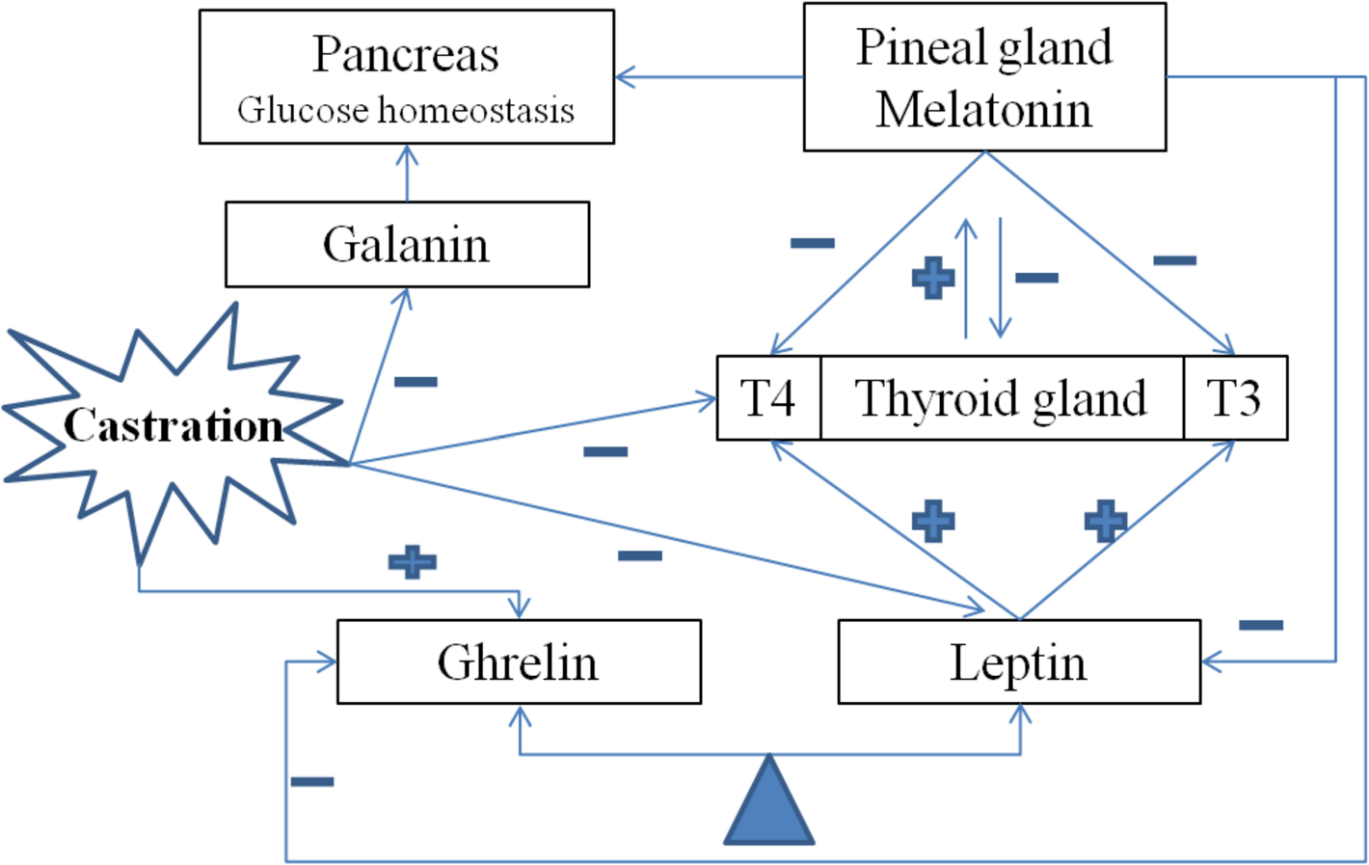
Schematic diagram of relationships between metabolic hormones (T3, T4. FT3, FT4, Leptin, Ghrelin, Galanin). The effects of melatonin and castration on metabolic hormones displayed as positive or negative symbols. Also, reciprocal relationship between pineal and thyroid glands and “Leptin-Ghrelin Tango” relationships indicated at top and bottom of diagram

According to the results of this study, melatonin reduced plasma levels of thyroid hormones, leptin, ghrelin and galanin. According to other studies, leptin can increase T3 and T4 levels [19], there is a reciprocal relationship between melatonin and thyroid hormones [13] and the nature of interaction between ghrelin and leptin is tango-like [38]. The schematic view of interactions between hormones has been shown in Fig. 10. There is no literature conducted on thyroid hormones effect on leptin, ghrelin and galanin.

## Conclusions

In conclusion, we observed some metabolic hormonal changes following castration such as decreased T3, T4, leptin and galanin and increased ghrelin levels. Administration of melatonin decreased concentration of all of them. Treatment of castrated dogs with melatonin kept the level of ghrelin at the level of intact dogs but T3, T4, leptin and galanin concentrations were lower than intact dogs. These effects would be useful in control of some side effects seen following castration.

## Abbreviations

ANOVA: Analysis of Variance
ELISA: Enzyme-Linked Immunosorbent Assay
FT3: Tree Triiodothyronine
FT4: Free Thyroxine
IM: Intramuscular
IV: Intravenous
T3: Triiodothyronine
T4: Thyroxine
